# Persistent Hepatitis E Virus Genotype 4 Infection in a Child With Acute Lymphoblastic Leukemia

**DOI:** 10.5812/hepatmon.15618

**Published:** 2014-01-23

**Authors:** Yansheng Geng, Hongxin Zhang, Weijin Huang, Tim J Harrison, Kunjing Geng, Zhuo Li, Youchun Wang

**Affiliations:** 1 Health Science Center, Hebei University, Baoding, China; 2 Division of HIV/AIDS and Sex-Transmitted Virus Vaccines, National Institutes for Food and Drug Control, Tiantanxili, Beijing, China; 3 Division of Medicine, University College London Medical School, London, UK; 4 Baoding Hospital for Infectious Disease, Baoding, China; 5 Affiliate Beijing You’an Hospital, Capital University of Medical Sciences, Hepatitis Institute of Beijing Municipal Health Bureau, Beijing, China

**Keywords:** Hepatitis E virus, Precursor Cell Lymphoblastic Leukemia-Lymphoma, Chronic Hepatitis, Persistent Infection

## Abstract

**Introduction:**

In general, the hepatitis E virus (HEV) causes acute, self-limiting hepatitis. Prolonged and chronic infections caused by HEV genotype 3 have been found in some immunosuppressed patients in developed countries.

**Case Presentation:**

Here we report a Chinese boy with acute lymphoblastic leukemia, who developed hepatitis E during a period of intensive chemotherapy. Twenty months after the initial infection, HEV viremia was reappeared in the patient, with detectable anti-HEV IgM and IgG and modestly elevated serum transaminases. Sequence analysis of the viral RNAs revealed the reactivation of the HEV genotype 4d strain, indicating viral persistence in the patient.

**Conclusions:**

To our knowledge, this is the first chronic case confirmed by the prolonged presence of HEV RNA in china. It is also the first reported persistent hepatitis E infection caused by HEV genotype 4.

## 1. Introduction

Hepatitis E virus (HEV) infection is a major cause of acute hepatitis in developing countries, and is an emerging health problem in industrialized countries. HEV is a non-enveloped virus with a single stranded, positive-sense RNA genome, of approximately 7.2 kb. HEV belongs to the genus Hepevirus of the family Hepeviridae, and four genotypes have been recognized to infect humans (1). HEV genotypes 1 and 2 are restricted to humans, and are often associated with large outbreaks and epidemics in developing countries with poor sanitation conditions, whereas HEV genotypes 3 and 4 infect humans, pigs and several other mammalian species, and are responsible for sporadic cases of hepatitis E in both developing and developed countries (1).

HEV infection is generally asymptomatic and most commonly manifests as a self-limiting, acute hepatitis in immunocompetent individuals (2). Chronic hepatitis E in immunosuppressed persons has been reported since 2008 (3). Patients with hematological disorders, HIV infection, or undergoing immunosuppressive therapy after solid organ transplantation, are at risk of developing chronic hepatitis E. Recent evidence suggests that about 50% of the cases of acute hepatitis E in immunocompromised patients progress to chronic hepatitis, with rapid progression to cirrhosis (4). Chronic hepatitis E cases have thus far been reported in France, Germany and other European countries and Canada. However, these chronic infections have all been caused by HEV genotype 3, which occurs as a zoonosis in these areas (1). In China, there is a high frequency of HEV epidemics of acute infections, which occur at about 4% per year, as estimated by the prevalence of anti-HEV IgM and the spontaneous rise of anti-HEV IgG levels (5). HEV genotype 4 has been found to be responsible for most hospitalized HEV cases in the recent years (6). However, to date, no chronic hepatitis E cases have been reported in China. Moreover, chronic HEV infection has been described in immunosuppressed adults, but rarely in children. Here, we report a persistent infection by HEV genotype 4d in a Chinese boy with acute lymphoblastic leukemia.

## 2. Case Presentation

**Figure 1.**
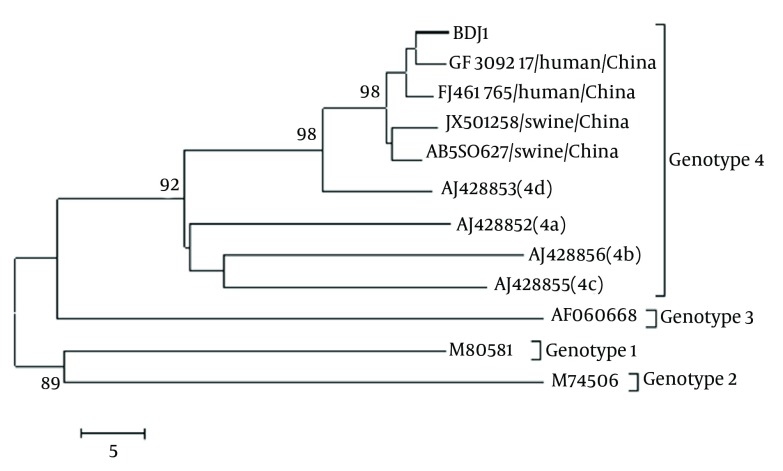
Phylogenetic Tree Constructed Based on Partial ORF2 Nucleotide Sequences (304 nt) by the Neighbor Joining Method Using MEGA 5.2 Statistical confidence for the tree was assessed by bootstrap analysis (the search option was 500 replications); bootstrap values greater than 85 were shown. The scale bar indicating the number of character state changes is proportional to the genetic distance. Database derived sequences are denoted by their GenBank accession numbers. The HEV isolate，BDJ1 (GenBank accession number, KF691589) from this study is indicated by a thick line.

A four-year-old boy was diagnosed with acute lymphoblastic leukemia in February 2011. Thereafter, he received a scheduled chemotherapy plan of 2 - 2.5 years. After cycles of chemotherapy, he received erythrocytes, platelets and fresh blood, due to chemotherapy-induced cytopenia. In early August, the boy complained of anorexia, nausea, and fatigue. Elevated levels of transaminases were detected in his serum. Serological tests for hepatitis A, B, C, and CMV had negative findings, whereas anti-HEV IgM was found to have positive results (Table 1). At admission on the 8th October, his serum values were as follows; alanine aminotransferase (ALT) 540 U/L (normal: < 45), aspartate aminotransferase (AST) 259 U/L (normal: < 50), total bilirubinemia (TBIL) 27.6μmol/L (normal: < 17.5 μmol/L), alkaline phosphatase (ALP) 157 U/L (normal: 45 - 135), c-glutamyltransferase (GGT) 97 U/L (normal: 10 - 47). Anti-HEV IgM anti-HEV IgG had positive results, but both were at low levels in terms of the ELSIA OD values of 0.27 and 0.22, respectively. HEV RNA was detected in the serum using RT-nested PCR. The HEV genotype was determined to be 4d by sequencing and analyzing the PCR amplicon (GenBank accession number: KF691589) (Figure 1). Physical examination at diagnosis of the HEV infection had normal findings, no jaundice or hepatosplenomegaly was observed. Color Doppler Ultrasound showed that the liver was normal in size and structure. However, when the patient insisted on leaving on November 10th for the following scheduled chemotherapy, HEV RNA in the serum had negative results, but the transaminases did not normalize; ALT 118 U/L, AST 94U/L, ACP 190 U/L, GGT 97 U/L.

In May 2013, twenty months later, the patient returned to our hospital for the treatment of hepatitis, after completing the latest intensive chemotherapy. Pathological values were obtained as follows; ALT 523 U/L, AST 822 U/L, ALP 527 U/L, GGT 352 U/L，serum albumin (ALB) 36.2 g/L (normal: 37-53 g/L), and TBIL 29.7 μmol/L. Anti-HEV IgM and IgG had positive results in the serum, and HEV RNA was also positive, with high levels up to 3.6 × 106 Geq/mL. The liver was still normal in size and structure by Color Doppler Ultrasound detection. The sequence of the 304 bp amplicon in the HEV ORF2 region was identical to that of the isolate obtained in 2011. It was most likely as a persistent HEV infection. Two months later, the HEV RNA levels had decreased in the serum to below detection limits, while the ALT level was still out of the normal range and anti-HEV IgM remained positive (Table 1). 

**Table 1.** Transaminases and Markers of HEV Infection in the Patient

Serum Collection Date	ALT ^a^, U/ L	AST ^a^, U/ L	Anti-HEV ^a^ IgM	Anti-HEV IgG	HEV-RNA, Geq/mL
**2011-08-02 ^b^**	376	146	Yes ^a^	No ^a^	NA ^a^
**2011-08-10 ** ^**b**^	241	150	Yes	No	NA
**2011-09-14 ** ^**b**^	585	263	Yes	No	NA
**2011-09-21 ** ^**b**^	166	60	NA	NA	NA
**2011-10-08**	540	259	Yes	Yes	3.2 × 10^2^
**2011-10-13**	331	135	NA	NA	NA
**2011-10-26**	128	95	Yes	Yes	3.2 × 10^2^
**2011-11-10**	118	94	Yes	Yes	No
**2013-05-11 ** ^**b**^	184	82	Yes	Yes	NA
**2013-05-21**	523	822	Yes	Yes	3.5 × 10^6^
**2013-06-04**	156	117	Yes	Yes	1.1 × 10^4^
**2013-07-20**	121	84	Yes	Yes	No

^a^ Abbreviations: ALT, alanine aminotransferase; AST, aspartate aminotransferase; HEV, hepatitis E virus, NA, not available; No, negative; Yes, positive.

^b^ The data was extracted from the clinical records transferred with the patient from another hospital.

## 3. Conclusions

The six-year-old boy described here, experienced prolonged HEV persistence for at least twenty months, with a modest elevation of transaminases during chemotherapy for acute lymphocytic leukemia. The identical viral sequences amplified from the patient at the two time points indicated the reactivation of endogenous HEV, but not re-infection. Chronic infection with hepatitis E virus has been reported in immunocompromised patients, including several patients with leukemia. Recurrence of HEV viremia was reported in an adult patient with leukemia after his acceptance for allogeneic stem cell transplantation, suggesting incomplete virus elimination due to immune deficiency (7). Chronic HEV infections have also been found in children with leukemia (8). HEV RNA was detected over 29 weeks in a 12-year-old boy presenting with early medullar relapse of lymphoblastic leukemia (9). The evolution of an HEV infection to chronic form seems to be related at least in part, to the intensity of the immunosuppressive therapy used. Indeed, reducing the dose of immunosuppressant given to patients can lead to the clearance of the virus (10). The case reported here is also in accordance with the earlier reports that chronic hepatitis E was associated with immunosuppression. Immunodeficiency due to both leukemia and long periods of chemotherapy exposed the boy at risk of a persistent HEV infection.

Chronic hepatitis E cases in immunocompromised patients have also been reported in both European countries and Canada. Such chronic infections have all been related to HEV genotype 3, which is prevalent in these areas. HEV is endemic in China, where HEV infection rates are generally higher than those reported in developed countries. In addition, the dominant HEV strains isolated in the recent years from sporadic human cases and pigs were genotype 4 (5, 11). However, until recently, no chronic hepatitis case was reported in China and no chronic case caused by genotype 4 has been reported in any other area. Phylogenetic analysis showed that the HEV strain detected from this patient belonged to subgenotype 4d. To our knowledge, this is the first case of a persistent hepatitis E infection caused by HEV genotype 4 and the first chronic case in China confirmed by the prolonged presence of HEV RNA. Since specific antibodies against the infected viruses may be absent or produced with delay in immunocompromised patients, serologic testing for the diagnosis of HEV infection is likely to be unreliable in this context, thus PCR-based detection of hepatitis E viral RNA is essential to make the diagnosis. Currently, clinical diagnosis of hepatitis E is primarily based on anti-HEV IgM detection in most hospitals. Therefore, chronic hepatitis E infection may be overlooked and misdiagnosed in cases where drug-induced liver injury is common in patients receiving chemotherapy or antiviral therapy. The prevalence of persistent hepatitis E infections in China and the effects of chronic hepatitis E in immunocompromised patients should be investigated.

The HEV genotype 4d strain detected in this patient showed high similarities with the genotype 4d strains isolated from humans and pigs in China, for instance the nucleotide similarities to the human strain GS-NJ-10 and swine strain HB-SB (GenBank accession Numbers: JF309217 and FJ461765) were 98.4% and 97%, respectively. However, the virus origin was difficult to clarify. The patient was in hospital for several months before the first HEV infection, thus, it is unlikely that he had been infected with HEV through direct contact with animals. The possibility of transmission via contaminated blood products could not be excluded, because the patient had received blood transfusions and blood products during the chemotherapy. In China, HEV-contaminated blood donations are a challenge for transfusion viral safety, since neither HEV antibodies nor HEV RNA are systematically tested in blood donors, and blood donations are currently not tested for ALT. Conversely, consumption of contaminated pork cannot be excluded completely in this patient, because pork is commonly consumed in China.
